# Antimicrobial resistance awareness and antibiotic prescribing behavior among healthcare workers in Nigeria: a national survey

**DOI:** 10.1186/s12879-020-05689-x

**Published:** 2021-01-07

**Authors:** Emelda E. Chukwu, David A. Oladele, Christian A. Enwuru, Peter L. Gogwan, Dennis Abuh, Rosemary A. Audu, Folasade T. Ogunsola

**Affiliations:** 1 Center for Infectious Diseases’ Research, Microbiology Department, Nigerian Institute of Medical Research, Yaba, Lagos, Lagos State Nigeria; 2 Clinical Science Department, Nigerian Institute of Medical Research, Yaba, Lagos, Lagos State Nigeria; 3 Center for Human Virology and Genomics, Microbiology Department, Nigerian Institute of Medical Research, Yaba, Lagos, Lagos State Nigeria; 4 Department of Medical Microbiology and Parasitology, College of Medicine, University of Lagos, Lagos, Nigeria

**Keywords:** Antibiotics, Antimicrobial resistance, Healthcare workers, Antibiotics prescription, Nigeria

## Abstract

**Background:**

Antimicrobial resistance (AMR) is a global problem compromising the effective treatment of infectious diseases. The World Health Organization (WHO) is encouraging and promoting awareness creation among health workers as one of its strategies to reduce the rate of emergence and transmission of AMR. Available data on the prescribing behavior of healthcare workers (HCWs) in Nigeria remains incomplete. This study was designed to provide an up-to-date estimate of the knowledge, attitude and antibiotic prescribing behavior of HCWs in Nigeria.

**Methods:**

This is a cross-sectional study. Self-administered questionnaires were distributed to healthcare workers selected from six states, one each from the 6 geopolitical zones in Nigeria. A multi-stage sampling technique was used to reflect the three tiers of healthcare: primary, secondary and tertiary levels. Quantitative data was summarized using descriptive statistics. All data analysis was done using the Statistical package for social sciences version 26.0.

**Results:**

Of the 420 questionnaires distributed, 358 (85.2%) responded. The mean year of practice of the respondents was 9.32 ± 7.8 years. About a half (50.3%) agreed that their prescribing behavior could promote antimicrobial resistance. 49.2% had a good knowledge of AMR and physicians had significantly better knowledge than other HCWs (X^2^ = 69.59, *P* < 0.001). Several participants prescribed antibiotics for common viral infections such as sore throats (75.7%), measles (37.7%), common cold and flu (21.2%). Over 60.3% admitted prescribing antibiotics just to be on the safe side. In general, 70.9% of the respondents frequently or moderately use practice guidelines while 25.7% often apply the delayed antibiotic prescription (DAP) strategy to reduce antimicrobial prescription.

**Conclusion:**

This study reveals an overall moderate level of knowledge of AMR and attitude towards minimizing the emergence of antimicrobial resistance though this did not translate significantly to practice. Further efforts must be made in order to improve rational prescription of antimicrobials among HCWs in Nigeria.

**Supplementary Information:**

The online version contains supplementary material available at 10.1186/s12879-020-05689-x.

## Introduction

Improving awareness and understanding of antimicrobial resistance (AMR) among the public, health professionals, policy makers and agricultural professionals is one of the key strategies of the global and national action plans to combat AMR and delay further emergence and spread of resistance [1]. However, emerging evidence on the impact of awareness raising on reducing antibiotic use remains inconclusive [1–4]. Awareness campaigns on the misuse of antibiotics for self-limiting conditions, such as coughs, colds, and viral upper respiratory infections (URI) are usually targeted at prescribers as well as at patients who demand antimicrobials [5, 6].

Delayed antibiotic prescription (DAP) is another strategy developed to reduce the inappropriate use of antibiotics in situations where the clinical indication is unclear [7]. Delayed prescribing is encouraged especially in primary care, and involves the prescription of antibiotics for a patient, to be taken only when the patient is not feeling better or feels worse several days after hospital visit [7, 8]. Nevertheless, this is not always implemented successfully. The non-prudent antimicrobial prescribing and dispensing practices in Low and Middle-Income Countries (LMICs) is often ascribed to a lack of knowledge about antimicrobial resistance and poor knowledge on the optimal use of antimicrobials, including antimalarials and antibiotics [9, 10]. However, some studies have argued that knowledge of antimicrobial resistance does not necessarily translate into a reduction in prescribing and dispensing of antimicrobials [7, 11–13]. The paucity of information on how healthcare professionals prescribe and dispense antimicrobials in LMICs is a significant research gap [14] and how this relates to AMR awareness should be understood in the Nigerian context, if successful strategies to contain AMR are to be developed and implemented.

Public healthcare services in Nigeria is organized into primary, secondary and tertiary levels. In 2005, it was estimated that 23,640 health facilities existed in Nigeria: 85.8% being primary health care facilities, 14% secondary and 0.2% tertiary care institutions [15]. The primary level of care is the usual entry point of communities into the health system and it typically provide general preventive, curative and pre-referral care [16]. Tertiary facilities provide the highest level of health care services including training of medical students and there is usually at least, one tertiary facility in every State, with a few having more than one [16].. Although only physicians, dentists and some nurses in primary care facilities have the legal right to prescribe medicines to patients in Nigeria, the allied medical personnel most often also prescribe drugs especially in primary healthcare facilities with very limited number of physicians [17]. More so, over-the-counter purchase of antimicrobials obtained without a prescription remains an issue in Nigeria [18].

An audit of a tertiary health centre in Lagos state found that more than 50% of antimicrobial agents prescribed to hospital patients were inappropriate, with cephalosporins being the most prescribed antibiotic. Unfortunately, microbiological testing was lacking and the most common test performed on these patients was a complete blood count [19]. Likewise, a survey of the specific prevalence of antimicrobial prescribing in four Nigerian tertiary hospitals by Oduyebo et al. [20], reported 69.7% for the prevalence rate of antimicrobial prescriptions with the highest prescription rates recorded in adult intensive care units (ICU) (88.9%). On the other hand, in a cross-sectional study of 105 physicians in Sokoto, north-western Nigeria, most physicians were unaware of the WHO’s global strategy for the containment of antimicrobial resistance and were also not up to date on information on antimicrobial resistance [21].

Prescribers often respond to patient expectations and requests [22]. Therefore, it is important to improve the understanding of patients and healthcare professionals on the appropriate use of antibiotics to achieve desired results. Increasing the proportion of physicians who adhere to guidelines for the prudent use of antibiotics is therefore a key strategy of the Global Plan of Action (GAP) to delay the onset of antimicrobial resistance. This study was designed to provide an up-to-date estimate of the knowledge, attitude and antibiotic prescribing behavior of HCWs in Nigeria. The current survey findings would inform research and guide development of strategies, intervention, and policies for the containment of antimicrobial resistance in Nigeria.

## Methods

### Study site and sampling

The study was carried out in six states (Jigawa Borno, Plateau, Ebonyi, Delta, and Lagos state) from each of the 6 geopolitical zones in Nigeria; North West, North East, North Central, South East, South South, and South West respectively. A multistage sampling technique was used to select the study participants. The first stage involved the selection of one state from each of the 6 geopolitical zones of the country through simple random sampling method. The second stage involved the selection of the health facilities. In each of the state chosen, one Tertiary Health Center, two Secondary Health Centers and three Primary Health Centers were selected from the list of all the health facilities in the state through a stratified random sampling technique taking into account the staff capacity of these health facilities as classified by the Nigerian demographic and Health Survey indices [23]. In stage three, study participants (different cadre of healthcare workers) were selected from a list of all healthcare workers through simple random sampling from each of the health facilities chosen in stage two. All healthcare workers who were either permanent staff or visiting consultants at the time of survey were eligible. Healthcare workers in administrative positions who are no longer involved in patient management were excluded from the study. The questionnaires were distributed over a period of 6 months from June–November 2019.

### Study design

This was a questionnaire-based observational study using a pre-tested questionnaire (Suppl 1). The questionnaire comprised 20 articles grouped into four sections: Section A (7 questions) obtained information on socio-demographics; section B (6 questions) was on the awareness of antimicrobial resistance. Question #11 in this section comprised a group of 8 questions which were used to assess the respondent’s knowledge level. Section C contained 4 questions which obtained information on the prescribing behavior, while section D (3 questions) asked about the use of guidelines and sources of information on AMR. The data collection tool was designed by reviewing relevant literatures and questionnaires previously used in similar studies [21, 24–26]. The questionnaire, which was anonymous and in English language, was pre-tested by administering to 20 medical practitioners working at the Nigerian Institute of Medical Research (NIMR) in order to determine the validity. The participants were asked to give their feedback and the level of comprehension of the questionnaire communication style and this was used to modify the questionnaire. These set of participants were not included in the survey. The validated questionnaires were randomly administered to the healthcare workers during a face-to-face visit to the selected healthcare facilities in the six geopolitical zones in Nigeria to reflect the primary (PHC), secondary (SHC) and tertiary health care centers (THC).

The eight knowledge questions were scored one for each correct answer and incorrect answers were scored zero with a maximum score of eight. The percentage distribution of the scores were calculated and used to classify respondents’ knowledge levels. Scores that were ≤ 25% were considered as poor AMR knowledge while scores ≥75% were indicative of good AMR knowledge. Respondents whose knowledge scores were greater than 25% but less than 75% were regarded to have fair or satisfactory knowledge. The questionnaires were self-administered, and respondents were allowed to retain the questionnaires if required while follow up visits for questionnaire mop-up was done over the period of study.

#### Statistical analysis

Quantitative data were summarized using descriptive statistics including frequencies, mean and standard deviation. All summed scores were assessed for normality using Shapiro-Wilk test for normality. Demographic characteristics that were significant for bivariate analysis were subjected to multivariate analysis to find out factors associated with the study outcome. Categorical variables were compared with chi-square test. Multivariate logistic regression analysis was used to model the odds of AMR relevance, antibiotic prescription influence and use of practice guideline across the study population. All data analysis was performed using SPSS window version 26.0 (version 26, IBM, Chicago, IL).

## Results

Out of the 420 questionnaires distributed 358 (85.2%) were filled and returned by the HCWs and analyzed. High level of non-response (30.7%) was recorded for SHC respondents. The respondents included 207 (57.8%) males and 151 (42.2%) females and were predominantly within the age range of 25 years to 44 years. The mean year of practice was 9.32 ± 7.8 years (95% CI 8.46–10.18) (Table 1).

**Table 1:** Demographic distribution of participants

Variable	Respondents	Percentage
**Gender**		
Male	207	57.8
Female	151	42.2
Age		
18–24	20	5.5
25–34	134	37.4
35–44	135	37.7
45–54	60	16.8
55–64	9	2.5
**Mean Year of practice ± STD 9.32 ± 7.8 (95% CI 8.46–10.18)Year of Practice**		
0–7	153	42.7
8–14	100	27.9
15–25	41	11.5
> 25	18	5.2
Missing	46	12.8
**Profession**		
Physicians	213	59.5
Nurse	63	17.6
Community health extension workers	35	9.8
Dentists	14	3.9
Pharmacists	6	1.7
Others (Allied health professionals)	27	7.5
**State**		
Borno	65	18.2
Delta	51	14.2
Ebonyi	63	17.6
Jigawa	62	17.3
Lagos	70	19.6
Plateau	47	13.1
**Health care center**		
Primary health care	63	17.6
Secondary health care	97	27.1
Tertiary health care	198	55.3
**How relevant is antimicrobial resistance for your daily work**		
Highly	190	53.1
Moderately	115	32.1
Sparsely	38	10.6
Not at all	15	4.2
**Use of Practice guideline for Antimicrobial therapy**		
Frequently	111	31
Moderately	143	39.9
Rarely	68	19
There are no good guidelines	16	4.5

### Awareness of antimicrobial resistance

Majority of the respondents (85.2%) believed that the subject of antimicrobial resistance was either highly or moderately relevant to their daily work (Table 1). Allied healthcare professionals (Others) were less likely to agree that the topic of antimicrobial resistance was relevant to their daily work (OR 0.29 95% CI 0.09–0.92, *p* = 0.035) (Table 2) while Healthcare workers in SHCs were more likely to believe that the subject of antimicrobial resistance was highly or moderately relevant to their daily work than their colleagues in THC (OR 2.94 95% CI 1.23–7.01, *p* = 0.015) (Table 2),

**Table 2:** Multivariate adjusted odd-ratios

**Question: How relevant is the subject of antibiotic resistance for your daily work?**		
**Answer: Highly/Moderately versus Sparsely/Not at all**		
**Variables**	**Odds ratio (95% Confidence Interval**	***P*-value**
**Profession**		
Physicians	Reference	
Nurses	0.96 (0.19–4.95)	0.965
Community health extension workers	0.51 (0.17–1.56)	0.239
Dentists	0.65 (0.13–3.14)	0.591
Pharmacists	0.44 (0.04–5.27)	0.52
Others	0.29 (0.09–0.92)	0.035
**State**		
Borno	Reference	
Delta	1.31 (0.39–4.37)	0.662
Ebonyi	4.69 (0. 93–23.74)	0.061
Jigawa	0.52 (0.16–1.68)	0.272
Lagos	0.53 (0.20–1.43)	0.211
Plateau	1.85 (0.51–6.72)	0.352
**Health care facility**		
Primary	1.32 (0.41–4.27)	0.649
Secondary	2.94 (1.23–7.01)	0.015
Tertiary	Reference	
**Question: Do you believe that your prescribing behavior influences the antibiotic resistance development within your region?**		
**Answer: Yes, Versus No or I don’t know**		
**Variables**	**Odds ratio (95% Confidence Interval**	***P*-value**
**Profession**		
Physicians	Reference	
Nurses	1.19 (0.37–3.82)	0.771
Community health extension workers	0.55 (0.25–1.24)	0.150
Dentists	0.71 (0.22–2.37)	0.581
Pharmacists	4.28 (0.46–40.14)	0.203
Others	0.43 (0.17–1.12)	0.083
**State**		
Borno	Reference	
Delta	1.21 (0.54–2.71)	0.644
Ebonyi	1.04 (0.49–2.22)	0.912
Jigawa	1.11 (0.45–2.75)	0.814
Lagos	1.56 (0.76–3.22)	0.225
Plateau	1.15 (0.51–2.56)	0.743
**Health care facility**		
Primary	0.32 (0.12–0.83)	0.020
Secondary	0.73 (0.43–1.25)	0.249
Tertiary	Reference	
**Question: Do you use practice guidelines for antibiotic therapy during your daily work?**		
**Answer: Frequently/Moderately versus Rarely/No good guideline**		
**Variables**	**Odds ratio (95% Confidence Interval**	***P*-value**
**Profession**		
Physicians	Reference	
Nurses	0.45 (0.14–1.47)	0.185
Community health extension workers	0.55 (0.24–1.27)	0.159
Dentists	1.40 (0.38–5.09)	0.613
Pharmacists	0.31 (0.53–1.76)	0.185
Others	0.47 (0.18–1.21)	0.119
**State**		
Borno	Reference	
Delta	1.31 (0.54–3.18)	0.553
Ebonyi	0.89 (0.40–1.99)	0.789
Jigawa	0.99 (0.39–2.58)	0.999
Lagos	1.89 (0.80–4.48)	0.146
Plateau	0.674 (0.29–1.55)	0.354
**Health care facility**		
Primary	1.03 (0.39–2.68)	0.945
Secondary	1.17 (0.64–2.12)	0.613
Tertiary	Reference	

**Fig. 1:**
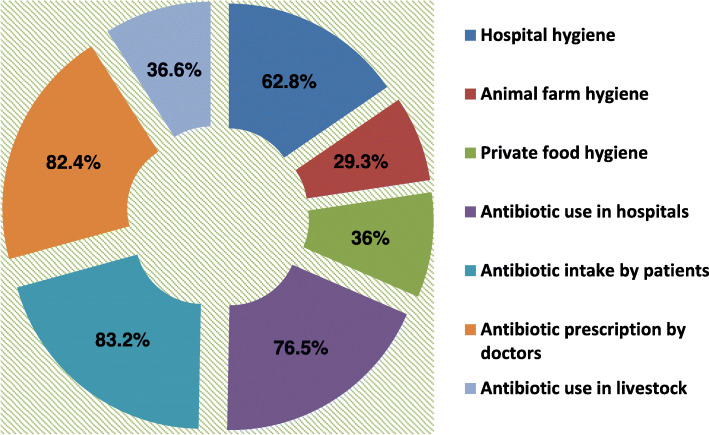
Participants response (%) to sectors to be targeted to slow down the emergence of AMR

When assessed for the level of knowledge on AMR, 176 (49.2%) and 169(47.2%) had good and fair knowledge respectively while 13(3.6%) had poor knowledge. Only 17% of the participants were able to answer all the eight questions correctly. Physicians had significantly better knowledge of AMR than their counterparts (X^2^ = 69.59, *P* < 0.001) (Table 3). There were variations among states with respondents from Lagos (62.9%) and Borno (61.5%) States more knowledgeable than those from Ebonyi (50.8%), Plateau (44.7%), Delta (41.2%) and Jigawa (29%) (X^2^ = 28.68, *P* = 0.001) (Table 3). Healthcare worker in the tertiary health care centers had better knowledge of AMR than their colleagues in the PHC and SHC (X^2^ = 24.99, *P* < 0.001) (Table 3). Using a multi-selection approach, majority of the respondents identified Antibiotic intake by patients (83.2%) and Antibiotic prescription by doctors (82.4%) as the major sectors to be targeted to slow down the development of AMR (Fig. 1).

**Table 3:** Distribution of knowledge score across the different variables

Variable	Knowledge Score				
	Poor (%)	Fair (%)	Good (%)	Chi-squared (X^**2**^)	***P***-value
**Gender**					
Male	5 (2.4)	89 (43.0)	113 (54.6)	6.78	0.034
Female	8 (5.3)	80 (53.0)	63 (41.7)		
**Age**					
18–24	2 (10)	8 (40)	10 (50)	14.48	0.152
25–34	7 (5.2)	54 (40.3)	73 (54.5)		
35–44	3 (2.2)	72 (53.3)	60 (44.4)		
45–54	1 (1.7)	33 (55.0)	26 (43.3)		
55–64	0 (0)	2 (22.2)	7 (77.8)		
**Profession**					
Physicians	1 (0.5)	75 (35.2)	137 (64.3)	69.59	< 0.001
Nurse	5 (7.9)	41 (65.1)	17 (27.0)		
Community health workers	1 (2.9)	29 (82.9)	5 (14.3)		
Dentist	1 (7.1)	6 (42.9)	7 (50.0)		
Others	5 (15.2)	18 (54.5)	10 (30.3)		
**Health care facility**					
Primary	6 (9.5)	42 (66.7)	15 (23.8)	24.99	< 0.001
Secondary	3 (3.1)	46 (47.4)	48 (49.5)		
Tertiary	4 (2.0)	81 (40.9)	113 (57.1)		
**States**					
Borno	1 (1.5)	24 (36.9)	40 (61.5)	28.68	0.001
Delta	2 (3.9)	28 (54.9)	21 (41.2)		
Ebonyi	0 (0)	31 (49.2)	32 (50.8)		
Jigawa	6 (9.7)	38 (61.3)	18 (29.0)		
Lagos	1 (1.4)	25 (35.7)	44 (62.9)		
Plateau	3 (6.4)	23 (48.9)	21 (44.7)		
Total	13 (3.6)	169 (47.2)	176 (49.2)

### Prescribing behavior

**Fig. 2:**
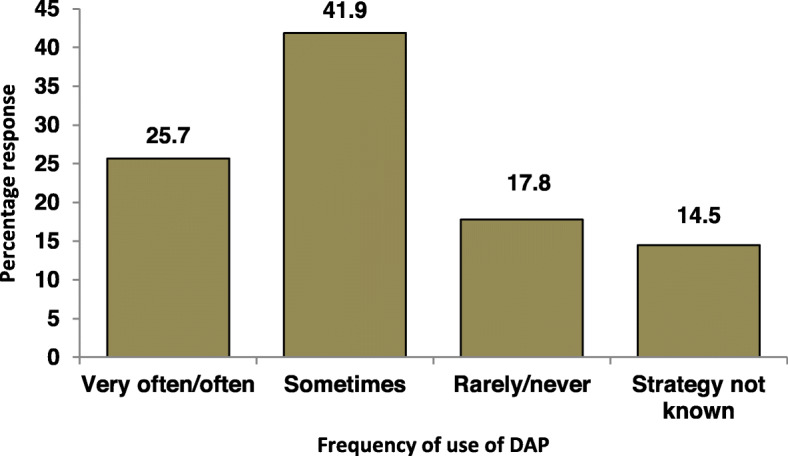
Participants response to the frequency of use of Delayed antibiotic prescribing (DAP) strategy

**Fig. 3:**
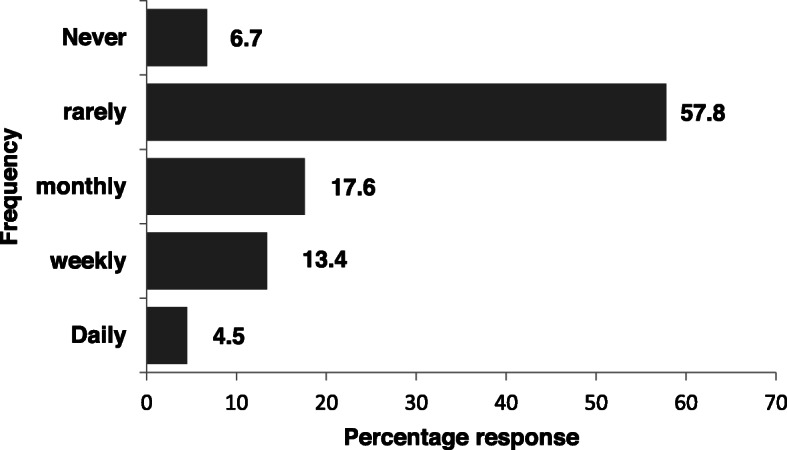
Frequency of contact with cases of Multi-drug resistant infections

About a half (50.3%) of the respondents understood that their prescribing behavior could promote the development of antimicrobial resistance while 36.0% and 13.7% believed otherwise or were not sure respectively. Lagos respondents were more aware that their prescribing behavior could promote the development of antimicrobial resistance than respondents from Borno although this was not statistically significant (OR 1.56 95%CI 0.76–3.2, *p* = 0.225) (Table 2). On the other hand, primary health care workers were less likely to assume individual antibiotic prescription influence on the development of drug resistance than their colleagues in the tertiary healthcare center (OR 0.32 95% CI 0.12–0.83, *p* = 0.02) (Table 2). However, only 25.7% often apply the DAP strategy to reduce antibiotics prescription (Fig. 2). Figure 3 shows their response to how often they encountered multi-drug resistant infections. A total of 35.5% of the respondents encountered multi-drug resistant infection at least every month (4.5% daily, 13.4% weekly and 17.6% monthly).

Some of the reason’s selected by respondents, why antibiotics maybe prescribed without indication in descending order are as follows;

- To be on the safe side (60.3%)
- When further diagnosis is too expensive for the patient (45.5%)
- When the cause of the disease is difficult to predict (36.0%)
- If patients demand an antibiotic (30.2%)
- If patient wants to get back to work quickly (29.9%)
- Cognitive impairment/ Language barrier (21.8%)
- If the patient is unresponsive (17.9%)
- none of the listed options (21.2%)

**Fig. 4:**
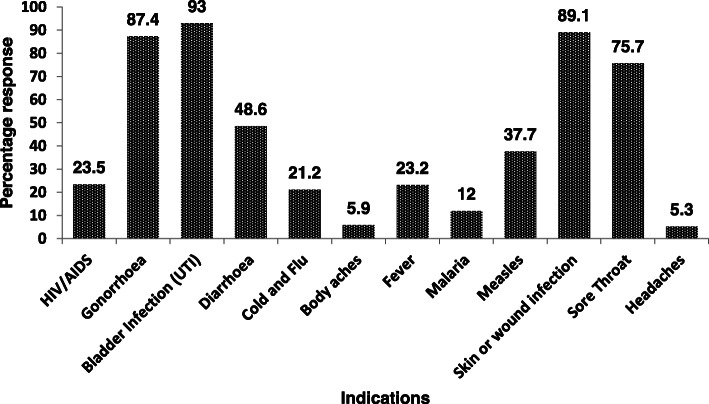
Participants depiction of Common Indications for antibiotics prescription

Majority of the respondents stated that the key barriers to discussing AMR with their patients is limited time for patient care (43.3%), lack of patient’s interest and difficulty in understanding (18.2%) and a perception that it may unsettle their patients (10.1%). For 13.4% of the respondents, a limited understanding prevents them from discussing AMR with their patients. 88.5% of respondents would perform hand hygiene, i.e. disinfect, or wash hands, as often as recommended and 94.1% agreed that antibiotics should only be prescribed when they are needed. Some of the respondents admitted to prescribing antibiotics for common viral infections including cold and flu (21.2%), measles (37.7%) and sore throats (75.7%) (Fig. 4).

### Use of guidelines / sources of information

**Fig. 5:**
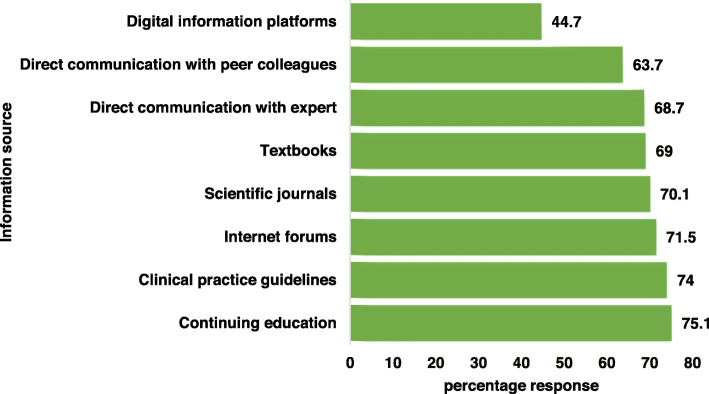
Sources of current information on antibiotic therapy and AMR

About 70.9% of the respondents use practice guideline either frequently (31.0%) or moderately (39.9%) (Table 1) and most (88.3%) of the respondents indicated that they would require a more evidence-based therapy guideline. The most mentioned sources of current information on antibiotic therapy and antimicrobial resistance cited by the participants in order of frequency comprised continuing education (75.1%), clinical practice guideline (74.0%), internet forums (71.5%), scientific journals (70.1%) and textbooks (69.0%) (Fig. 5). The odds of using practice guideline was higher in HCWs in Lagos state than their counterparts in Borno State (OR 1.89 95% CI 0.80–4.48, *p* = 0.146) (Table 2).

## Discussion

We conducted a questionnaire-based survey of the level of awareness of healthcare workers on rising antimicrobial resistance, patient communication, prescribing behavior, and the source of information on AMR in Nigeria. The awareness of antimicrobial resistance among medical practitioners has risen during the past few years and has been demonstrated by studies across the globe [12, 25, 26]. There was an overall fair knowledge of AMR among our participants although this was skewed more towards the physicians. However, this did not translate to reduced antimicrobial prescription since a substantial number of our respondents prescribed antibiotics for common viral infections including sore throats, measles as well as cold and flu. A recent study had challenged the assumption that *injudicious prescription practices* among HCWs is as a result of low levels of awareness of AMR and poor knowledge of the optimal use of antimicrobials. The authors reported high AMR awareness among professionals across seven LMIC settings and suggested that addressing contextual restraints will be as important as raising awareness in tackling AMR [12].

Practitioners in the THC had better knowledge of AMR than their colleagues in PHC and SHC and this was significantly linked to assuming individual responsibility for their prescription behavior influence on the development of AMR. There were significant disparities in the responses to questions on prescribing behavior across the healthcare centers with respondents in PHCs less likely to agree that their prescribing behavior can influence the development of antimicrobial resistance. These specific differences need to be considered in tailoring of interventions to ensure a wholistic approach is adopted. The higher level of awareness in the THCs may likely relate to the fact that this sector had greater proportion of physicians. Nonetheless, this trend should be examined and effort should be made to improve the knowledge level and prescription behavior of the HCWs in the PHCs and SHCs which are most likely to be the first point of call for most patients and the prospective foundation for the development of AMR [16, 17]. We note that majority of respondents would perform hand hygiene, i.e. disinfect or wash hands, in line with WHO recommendations on hand hygiene in health care system [27] and this is critical because, poor infection prevention and control measures has been listed as one of the key factors that accelerates the transmission of AMR [22].

A previous study, lamented high antimicrobial prescription rate in the tertiary healthcare facilities in Nigeria and the authors concluded that there is need to create awareness at the national level for targeted prescribing of antimicrobials and use of evidence-based guidelines [20]. Four years down the line, there seem to be minimal improvement with regards to the use of guideline and prudent use of antimicrobials with only 31% of our respondents indicating that they often use the practice guideline. This may be attributed to the poor access to available guidelines and the general lack of antimicrobial stewardship in most of the health facilities in Nigeria [28]. Improvement in the use of guideline in daily outpatient care has become very essential, considering that 35.5% of our respondents encounter a multi-drug resistant infection at least every month.

Delay prescription strategy is an alternative and effective way to reduce antibiotic use and invariably delay emergence of antimicrobial resistance especially in respiratory tract infections [8, 29]. However, 14.5% of respondents in our study were not aware of the strategy while 17.8% rarely or never use it. This result is consistent with the report from a similar study in Germany where only 29.4% of the respondents applied the strategy out of 99.1% who had knowledge of the strategy [25]. Similarly, a qualitative study in England suggested that greater uniformity within and between practices is needed to operationalize delayed prescribing, as well as providing feedback on the uptake of antibiotics [7].

This study demonstrates different specific barriers to rational antimicrobial therapy in health care centers in Nigeria. Worthy of note is that 60.3% of the respondents admitted to prescribing antibiotics just to be on the safe side. This is usually done without the use of diagnostic tools to guide prescription. In addition, about one-third (30.2%) of our respondents will prescribe antimicrobial when demanded by patient. This trend has also been reported in other studies with some authors emphasizing that when general practitioners feel pressure from their patients, they are more likely to prescribe antimicrobials [29–31]. Our finding may be lending credence to a previous report stating that more than 50% of antimicrobial agents prescribed in a tertiary hospital in Lagos State, Nigeria for inpatients were inappropriate [19].

On the aspect of communication, majority of the respondents cited *lack of time* (43.3%) and *Lack of patient’s interest* (18.2%) as a reason for not discussing AMR with their patients suffering from infections while prescribing antibiotics for them. This pattern has also been reported in other countries [25, 26]. The listed barrier to AMR communication came as no surprise because most of these healthcare facilities are short staffed and would rather reduce patient time to the barest minimum.

Registered health workforce profile released in 2013, showed there were 65,759 medical doctors, 249,566 nurses and midwives, 16, 979 pharmacists, 1849 pharmacy technicians, as well as 71,396 Community Health Extension Workers in Nigeria [17]. The uneven distribution of health workers is evident by cadre as physician specialists are more in the tertiary and secondary health care facilities while community health extension workers are more in the primary healthcare centers where they provide the majority of the healthcare services [17]. Our study noted that most of the primary health facilities suffered inadequate number of physicians and as a result engage the services of nurses and community health extension workers for patient management who also prescribe antimicrobials when indicated (Suppl2 Table 2). Unfortunately, these healthcare professionals were less likely to agree that the topic of antimicrobial resistance was either highly or moderately relevant to their daily work. There is therefore need to ensure that these cadre of health care workers are properly trained on the prescription guideline, and future antimicrobial stewardship programs should include these cadre of health workers. Antimicrobial stewardship program (ASP) ideology advocated by the WHO has not enjoyed widespread application in healthcare facilities in Nigeria [28]. The adoption of antimicrobial stewardship by healthcare providers involves the use of objective interventions to influence prescribing practices, thereby promoting rationale and appropriate use of antimicrobials [32, 33]. A recent study revealed that only about a third (35%) of the tertiary health facilities sampled across the Nigeria had a formal organizational structure and a team responsible for ASP [28]. Furthermore, an even lower percentage of the health facilities (24%) had in place, a facility-specific treatment recommendation based on the local AMR patterns. This poor level of antimicrobial stewardship needs to be addressed before any meaningful progress can be made in the fight against emergence of antimicrobial resistance in Nigeria.

This study had certain limitations. As a questionnaire-based survey, there was the risk that respondents would want to give answers believed to be socially correct rather than their genuine opinion. In addition, this research did not address AMR awareness and antimicrobial use among registered practitioners working in private health facilities who are known to play a prominent role in the provision of antimicrobials across many LMIC settings [34]. Another limitation is that selection of one state per geopolitical zone may provide limited representative data of the entire zone.

Some of the perceived strength of the study is that the use of stratified random sampling allowed for the inclusion of healthcare practitioners working in the primary health centers in remote areas where there is limited number of medical doctors and poor access to information. Also, the use of knowledge questions to assess the level of knowledge ensured the avoidance of bias from self-knowledge scoring. Unlike previous studies which have focused mainly on the physicians [21], inclusion of other HCWs aside from the physicians in this study allowed for a wider insight on the level of awareness on AMR especially among HCWs in primary health centers where there are limited physicians. There is need to adopt a wholistic approach when creating awareness on AMR to include all cadre of HCWs involved in the treatment and management of patients.

## Conclusion

This study reveals that the knowledge of antimicrobial resistance is fair among healthcare workers in Nigeria. However, measures to improve rational prescription are not widely implemented yet. It is important to note that the survey showed wide variation on the knowledge and antimicrobial prescription behavior across the healthcare centers and states. State/healthcare center specific strategies and actions are therefore encouraged. This could be achieved through increased campaigning efforts, advisory and policy documents, stewardship programs as well as establishment of surveillance system for monitoring AMR. This would be useful to determine current trends and help with HCW support for best practice guidelines. In addition, there is need for continuous education and training of HCWs through healthcare worker-specific engagements, and the development of interventions informed by behavior change theories and models.

## Supplementary Information

**Additional file 1: **Questionnaire**.**

**Additional file 2: Table 1**: Across and within state distribution of knowledge score. **Table 2**: Distribution of Healthcare workers in the different States. **Figure 1**: Summary of responses by State Healthcare workers to the relevance of the topic of Antimicrobial resistance to their daily work. **Figure 2**: Summary of responses by State healthcare to the influence of their Antimicrobial prescription behavior to the development of antimicrobial resistance. **Figure 3**: Across the profession response to the relevance of Antimicrobial resistance to daily work. **Figure 4**: Across the profession response to the influence of Antimicrobial prescribing behavior to the development of AMR. **Figure 5**: Across the healthcare center response to relevance of the topic of Antimicrobial resistance to their daily work. **Figure 6**: Across the healthcare center response to the influence of their Antimicrobial prescription behavior to development of antimicrobial resistance

## Data Availability

All data generated or analyzed during this study are included in this published article [and its supplementary information files] Original data are available from the corresponding author upon reasonable request.

## Abbreviations

AMR: Antimicrobial resistance
CI: Confidence interval
OR: Odds ratio
HCW: Healthcare workers
ASP: Antimicrobial resistance stewardship program
LMIC: Low and middle income countries
DAP: Delayed antibiotic prescription
WHO: World health Organization
NIMR: Nigerian institute of medical research
FETHA: Federal teaching hospital Abakaliki
IRB: Institutional review board
PHC: Primary healthcare center
SHC: Secondary healthcare center
THC: Tertiary healthcare center
ICU: Intensive care unit
SPSS: Statistical software for social sciences
SD: Standard deviation
