# Supplementary material for: Antimicrobial resistance awareness and antibiotic prescribing behavior among healthcare workers in Nigeria: a national survey
Source: BMC Infect Dis. 2021 Jan 7;21:22. doi: 10.1186/s12879-020-05689-x (PMC7792030; doi:10.1186/s12879-020-05689-x)
Supplement: Supplementary file 1 — Additional file 1: Questionnaire. [file 12879_2020_5689_MOESM1_ESM.docx]

**QUESTIONNAIRE**

**A national survey of Antibiotic prescribing behaviour among**

**Medical practitioners in Nigeria**

**SECTION A: Socio demographic characteristics of respondents**

1. Address/ location ………………………………………………………………………

2. State ……………………………………………………………

3. Year of practice ………………………

4. Profession: Medical Doctor Nurses Dentist Pharmacist

Others, specify………………………….

5. Gender: Male Female

**6. What age range do you belong?**  18-24 25-34 35-44

45-54 55-64 65+

**7. Type of health care center:** Primary health care Secondary healthcare Tertiary/ Referral center

**SECTION B: AWARENESS OF ANTIMICROBIAL RESISTANCE**

**8.** **How relevant is the subject of antibiotic resistance for your daily work?**

Highly moderately sparsely not at all

**9. How often do you have contact to patients with Multi-Drug Resistant infection during your daily practice?**

Daily weekly monthly rarer never

**10. What do you think should be the sectors to be targeted to slow down the development of antibiotic resistances?**

Tick all that applies

| Hospital hygiene |  |
| --- | --- |
| Animal farm hygiene |  |
| Private food hygiene |  |
| Antibiotic use in hospitals |  |
| Antibiotic intake by patients |  |
| Antibiotic prescriptions by doctors |  |
| Antibiotic use in livestock |  |

**11.Please indicate if you think the following statements are ‘true’ or ‘false’ (write TorF)**

| a. Antibiotic resistance occurs when your body becomes resistant to antibiotics  and they no longer work as well |  |
| --- | --- |
| b. Many infections are becoming increasingly resistant to treatment by antibiotics |  |
| c. If bacteria are resistant to antibiotics, it can be extremely difficult or impossible to treat the infections they cause |  |
| d. Prescription of antibiotics with overly broad-spectrum coverage can precipitate Antibiotic resistance |  |
| e. Antibiotic resistance is an issue in other countries but not here |  |
| f. Antibiotic resistance is only a problem for people who take antibiotics regularly |  |
| g. Bacteria which are resistant to antibiotics can be spread from person to person |  |
| h. Antibiotic-resistant infections could make medical procedures like surgery, organ transplants and cancer treatment much more dangerous |  |

**12. On the scale shown, how much do you agree that the following actions would help address the problem of antibiotic resistance?**

|  |  | **Agree Strongly** | **Agree** | **Neither agree nor disagree** | **Disagree** | **Disagree strongly** |
| --- | --- | --- | --- | --- | --- | --- |
| **A** | Healthcare workers should wash their hands regularly (i.e. disinfect or wash hands, as often as recommended) |  |  |  |  |  |
| **B** | Prescribers should only prescribe antibiotics when they are needed |  |  |  |  |  |

**SECTION C: PRESCRIBING BEHAVIOR**

**13.** **Do you believe that your prescribing behaviour influences the antibiotic resistance development within your region?**

Yes No don’t know

**14. Do you use the strategy of delayed antibiotic prescribing?**

Strategy not known very often often

Sometimes rarely never

**15. Which of these to you are the reasons antibiotics maybe prescribed without an indication? Tick all that apply**

| When the weekend is approaching and the course of the disease is difficult to predict |  |
| --- | --- |
| If the patient wants to get back to work quickly |  |
| If the patient demands an antibiotic |  |
| If the patient is incompliant |  |
| Language barriers or cognitive impairments |  |
| Because further diagnostics are too expensive |  |
| To be on the safe side |  |
| None of the above (will not prescribe without a diagnosis) |  |

**16. Indications for an antibiotic prescription for me are? tick all that apply**

| HIV/AIDS |  | Fever |  |
| --- | --- | --- | --- |
| Gonorrhoea |  | Malaria |  |
| Bladder infection or urinary tract infection (UTI) |  | Measles |  |
| Diarrhoea |  | Skin or wound infection |  |
| Cold and flu |  | Sore throat |  |
| Body aches |  | Headaches |  |

**17. Barriers to discussing antimicrobial resistance (AMR) with patients while prescribing.**

Lack of time Concern that it will unsettle the patient

Lack of patients interest Limited knowledge about the subject

**SECTION D: GUIDELINES/SOURCES OF INFORMATION**

**18.** **Do you use practice guidelines for antibiotic therapy during your daily work?**

Frequently moderately rarely or never there are no good guidelines

**19. Would you like to have more evidence-based therapy guidelines?**

Yes no don’t know

**20. Which are your sources to get current information on** **antibiotic therapy and Antibiotic resistance? Select all that apply**

| Internet forums |  |
| --- | --- |
| Digital information platforms |  |
| Textbooks |  |
| Scientific journals |  |
| Clinical practice guidelines |  |
| Direct communication with peer colleagues |  |
| Direct communication with expert |  |
| Continuing education |  |
