# Supplementary material for: Antimicrobial resistance awareness and antibiotic prescribing behavior among healthcare workers in Nigeria: a national survey
Source: BMC Infect Dis. 2021 Jan 7;21:22. doi: 10.1186/s12879-020-05689-x (PMC7792030; doi:10.1186/s12879-020-05689-x)
Supplement: Supplementary file 2 — Additional file 2: Table 1: Across and within state distribution of knowledge score. Table 2: Distribution of Healthcare workers in the different States. Figure 1: Summary of responses by State Healthcare workers to the relevance of the topic of Antimicrobial resistance to their daily work. Figure 2: Summary of responses by State healthcare to the influence of their Antimicrobial prescription behavior to the development of antimicrobial resistance. Figure 3: Across the profession response to the relevance of Antimicrobial resistance to daily work. Figure 4: Across the profession response to the influence of Antimicrobial prescribing behavior to the development of AMR. Figure 5: Across the healthcare center response to relevance of the topic of Antimicrobial resistance to their daily work. Figure 6: Across the healthcare center response to the influence of their Antimicrobial prescription behavior to development of antimicrobial resistance [file 12879_2020_5689_MOESM2_ESM.docx]

**Supplementary material 2**

**Table 1: Across and within state distribution of knowledge score**

| **State** | | | **Knowledge score** | | | **Total** | **X^2^** | **P. value** |
| --- | --- | --- | --- | --- | --- | --- | --- | --- |
|  |  |  | **Poor (%)** | **Fair**  **(%)** | **Good**  **(%)** |  |  |  |
| **Borno** |  | Primary | 1(10.0) | 6(60.0) | 3(30.0) | 10 | 9.22 | 0.056 |
|  |  | Secondary | 0(0) | 6(30.0) | 14(70.0) | 20 |  |  |
|  |  | Tertiary | 0(0) | 12(34.3) | 23(65.7) | 35 |  |  |
|  | **Total** | | **1(1.5)** | **24(36.9)** | **40(61.5)** | **65** |  |  |
| **Delta** |  | Primary | 0(0) | 5(71.4) | 2(28.6) | 7 | 2.21 | 0.698 |
|  |  | Secondary | 0(0) | 8(57.1) | 6(42.9) | 14 |  |  |
|  |  | Tertiary | 2(6.7) | 15(50.0) | 13(43.3) | 30 |  |  |
|  | **Total** | | **2(3.9)** | **28(54.9)** | **21(41.2)** | **51** |  |  |
| **Ebonyi** |  | Primary | 0(0) | 8(80.0) | 2(20.0) | 10 | 11.93 | 0.003 |
|  |  | Secondary | 0(0) | 13(68.4) | 6(31.6) | 19 |  |  |
|  |  | Tertiary | 0(0) | 10(29.4) | 24(70.6) | 34 |  |  |
|  | **Total** | | **0(0)** | **31(49.2)** | **32(50.8)** | **63** |  |  |
| **Jigawa** |  | Primary | 2(20.0) | 5(50.0) | 3(30.0) | 10 | 1.82 | 0.768 |
|  |  | Secondary | 2(10.0) | 12(60.0) | 6(30.0) | 20 |  |  |
|  |  | Tertiary | 2(6.2) | 21(65.6) | 9(28.1) | 32 |  |  |
|  | **Total** | | **6(9.7)** | **38(61.3)** | **18(29.0)** | **62** |  |  |
| **Lagos** |  | Primary | 1(7.7) | 8(61.5) | 4(30.8) | 13 | 10.6 | 0.031 |
|  |  | Secondary | 0(0) | 7(36.8) | 12(63.2) | 19 |  |  |
|  |  | Tertiary | 0(0) | 10(26.3) | 28(73.7) | 38 |  |  |
|  | **Total** | | **1(1.4)** | **25(35.7)** | **44(62.9)** | **70** |  |  |
| **Plateau** |  | Primary | 2(15.4) | 10(76.9) | 1(7.7) | 13 | 15.67 | 0.003 |
|  |  | Secondary | 1(20.0) | 0(0) | 4(80.0) | 5 |  |  |
|  |  | Tertiary | 0(0) | 13(44.8) | 16(55.2) | 29 |  |  |
|  | **Total** | | **3(6.4)** | **23(48.9)** | **21(44.7)** | **47** |  |  |
| **Total** |  | Primary | 6(9.5) | 42(66.7) | 15(23.8) | 63 | 24.993 | <0.001 |
|  |  | Secondary | 3(3.1) | 46(47.4) | 48(49.5) | 97 |  |  |
|  |  | Tertiary | 4(2.0) | 81(40.9) | 113(57.1) | 198 |  |  |
|  | **Total** | | **13(3.6)** | **169(47.2)** | **176(49.2)** | **358** |  |  |

| **Table 2: Distribution of Healthcare workers in the different States** | | | | | | | | | |
| --- | --- | --- | --- | --- | --- | --- | --- | --- | --- |
|  | | | | | | | | | |
| Health care Centre | | | State | | | | | | Total |
|  |  |  | Borno | Delta | Ebonyi | JIgawa | Lagos | Plateau |  |
| Primary healthcare center | Profession | Physicians | 3 | 0 | 0 | 0 | 3 | 0 | 6 |
|  |  | Dentists | 0 | 0 | 0 | 0 | 1 | 0 | 1 |
|  |  | Nurse | 4 | 4 | 6 | 1 | 0 | 3 | 18 |
|  |  | Community health workers | 1 | 3 | 4 | 8 | 8 | 8 | 32 |
|  |  | Others | 2 | 0 | 0 | 1 | 1 | 2 | 6 |
|  | Total | | 10 | 7 | 10 | 10 | 13 | 13 | 63 |
| Secondary healthcare center | Profession | Physicians | 20 | 9 | 3 | 1 | 16 | 3 | 52 |
|  |  | Dentists | 0 | 0 | 1 | 0 | 3 | 0 | 4 |
|  |  | Nurse | 0 | 2 | 11 | 11 | 0 | 2 | 26 |
|  |  | Community health workers | 0 | 0 | 1 | 2 | 0 | 0 | 3 |
|  |  | Pharmacists | 0 | 2 | 1 | 2 | 0 | 0 | 5 |
|  |  | Others | 0 | 1 | 2 | 4 | 0 | 0 | 7 |
|  | Total | | 20 | 14 | 19 | 20 | 19 | 5 | 97 |
| Tertiary healthcare center | Profession | Physicians | 34 | 18 | 32 | 8 | 34 | 29 | 155 |
|  |  | Dentists | 0 | 2 | 2 | 1 | 4 | 0 | 9 |
|  |  | Nurse | 0 | 0 | 0 | 19 | 0 | 0 | 19 |
|  |  | Pharmacists | 0 | 0 | 0 | 1 | 0 | 0 | 1 |
|  |  | Others | 1 | 10 | 0 | 3 | 0 | 0 | 14 |
|  | Total | | 35 | 30 | 34 | 32 | 38 | 29 | 198 |
| Total | Profession | Physicians | 57 | 27 | 35 | 9 | 53 | 32 | 213 |
|  |  | Dentists | 0 | 2 | 3 | 1 | 8 | 0 | 14 |
|  |  | Nurse | 4 | 6 | 17 | 31 | 0 | 5 | 63 |
|  |  | Community health workers | 1 | 3 | 5 | 10 | 8 | 8 | 35 |
|  |  | Pharmacists | 0 | 2 | 1 | 3 | 0 | 0 | 6 |
|  |  | Others | 3 | 11 | 2 | 8 | 1 | 2 | 27 |
|  | Total | | 65 | 51 | 63 | 62 | 70 | 47 | 358 |

**Figure 1: Summary of responses by State Healthcare workers Figure 2: Summary of responses by State healthcare workers to the relevance of the topic of Antimicrobial resistance to the influence of their Antimicrobial prescription to to their daily work behavior to the development of antimicrobial resistance**

**Figure 3: Across the profession response to the relevance of Figure 4: Across the profession response to the influence of**

**Antimicrobial resistance to daily work Antimicrobial prescribing behavior to the development of**

**AMR**

**Figure 5: Across the healthcare center response to Figure 6: Across the healthcare center response to the influence**

**relevance of the topic of Antimicrobial resistance to their of their Antimicrobial prescription behavior to development**

**daily work of antimicrobial resistance**
